# Ubiquitin-Specific Protease 4 Inhibits Mono-Ubiquitination of the Master Growth Factor Signaling Kinase PDK1

**DOI:** 10.1371/journal.pone.0031003

**Published:** 2012-02-07

**Authors:** Iris Z. Uras, Thomas List, Sebastian M. B. Nijman

**Affiliations:** CeMM – Research Center for Molecular Medicine of the Austrian Academy of Sciences, Vienna, Austria; University of Hong Kong, Hong Kong

## Abstract

**Background:**

Phosphorylation by the phospho-inositide-dependent kinase 1 (PDK1) is essential for many growth factor-activated kinases and thus plays a critical role in various processes such as cell proliferation and metabolism. However, the mechanisms that control PDK1 have not been fully explored and this is of great importance as interfering with PDK1 signaling may be useful to treat diseases, including cancer and diabetes.

**Methodology/Principal Findings:**

In human cells, few mono-ubiquitinated proteins have been described but in all cases this post-translational modification has a key regulatory function. Unexpectedly, we find that PDK1 is mono-ubiquitinated in a variety of human cell lines, indicating that PDK1 ubiquitination is a common and regulated process. Ubiquitination occurs in the kinase domain of PDK1 yet is independent of its kinase activity. By screening a library of ubiquitin proteases, we further identify the Ubiquitin-Specific Protease 4 (USP4) as an enzyme that removes ubiquitin from PDK1 *in vivo* and *in vitro* and co-localizes with PDK1 at the plasma membrane when the two proteins are overexpressed, indicating direct deubiquitination.

**Conclusions:**

The regulated mono-ubiquitination of PDK1 provides an unanticipated layer of complexity in this central signaling network and offers potential novel avenues for drug discovery.

## Introduction

Growth factors such as insulin promote fundamental cellular processes such as proliferation and survival through the activation of signaling cascades involving numerous protein kinases. Of these, the phosphoinositide-3 kinase (PI3K) and the phosphoinositide-dependent kinase 1 (PDK1) play a critical role [1]. Upon activation, PI3K generates second messenger molecules consisting of phosphoinositides that prime members of the AGC superfamily of protein kinases for activation. At least twenty-three of the AGC kinases require phosphorylation by PDK1 at a conserved residue in their activation loop (also called T-loop), including central enzymes such as the proto-oncogene AKT, Protein Kinase C (PKC) and the p70 S6 kinase (S6K) [2], [3], [4], [5]. Together, these and other downstream kinases coordinate cell growth, proliferation, survival and metabolism and they are frequently found deregulated in many diseases such as cancer and diabetes [6], [7], [8], [9].

The mechanisms that keep PDK1 activity in check are not fully investigated. Yet, these are of great interest as the ability to interfere with the activation of its target kinases would be of great therapeutic importance. Given that PDK1 is such an important kinase, it is remarkable that it is found constitutively active due to *in trans* autophosphorylation of its T-loop residue [10]. Indeed, regulation of substrate accessibility is thought to be a major mechanism whereby PDK1 activity is controlled. In the case of AKT, this is achieved by recruitment to phospholipids generated by PI3K at the plasma membrane via the Pleckstrin Homology (PH) domains of both kinases. For other AGC kinases such as S6K and serum- and glucocorticoid-induced kinase (SGK), a priming phosphorylation on the hydrophobic motif by other kinases stimulates interaction with the PDK1 interacting fragment (PIF) pocket near the catalytic domain of PDK1, thereby facilitating T-loop phosphorylation [5], [11], [12]. PDK1 has also been described to shuttle between the nucleus and the cytoplasm in a growth factor dependent manner but the significance of this in terms of target activation has not been addressed [13], [14]. Given the central role of PDK1 in the regulation of many downstream effectors, it is likely that additional regulatory mechanisms remain to be discovered.

The addition of the small molecule ubiquitin to proteins plays a critical role in essentially all biological processes. Indeed, defects in this control mechanism can cause many diseases including cancer [15], [16], [17]. The addition of polyubiquitin chains to proteins was originally identified as a mechanism for targeting proteins to the 26S proteasome for degradation. However, it has recently been shown that selective mono-ubiquitination or alternative ubiquitin chains can also regulate protein activity [18], [19], [20], [21], [22], [23], [24]. These non-proteolytic functions of ubiquitination play diverse roles in DNA damage repair, protein trafficking and localization and activation of signal transduction pathways. Like phosphorylation, ubiquitination is reversible and mediated by deubiquitinating enzymes (DUBs) that cleave the isopeptide bond at the carboxy terminus of ubiquitin [25]. Furthermore, DUBs have also become actively studied drug targets for cancer therapy [26], [27].

Various PI3K pathway components are regulated by ubiquitination, including AKT and PTEN [28], [29], [30]. However, for PDK1 no post-translational modification other than phosphorylation has been described. Here, we show that PDK1 is mono-ubiquitinated and that this modification occurs in the amino-terminal kinase domain. In addition, a cDNA library consisting of DUBs was screened for novel regulators of PDK1 ubiquitination and USP4 was identified as a potential modulator of PDK1 ubiquitination.

## Results

### PDK1 is modified by mono-ubiquitination

**Figure 1:**
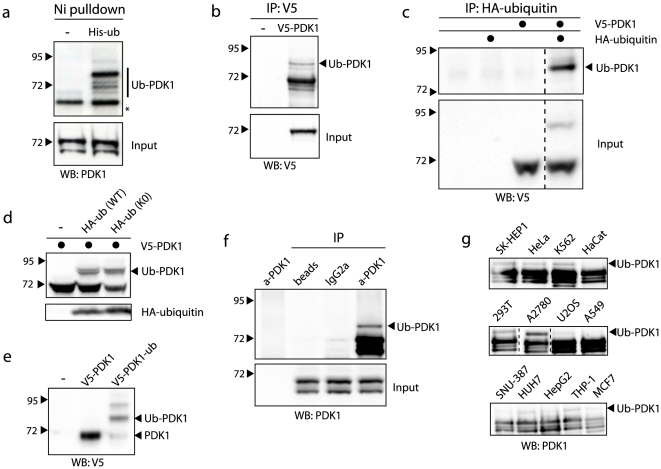
PDK1 is mono-ubiquitinated in a variety of cell lines. **A**: HEK293T cells were transfected with 6×His-tagged ubiquitin and ubiquitinated proteins were isolated with nickel beads under denaturing conditions. Pull-downs and input were analyzed with an antibody recognizing PDK1. Ub-PDK1 indicates ubiquitin-modified PDK1 species; asterisk indicates unspecific cross-reacting band. **B**: HEK293T cells were transfected with a V5-tagged PDK1 cDNA and V5-PDK1 was immunoprecipitated. Immunoprecipitations (IP) and input were immunoblotted with a V5 antibody. **C**: HEK293T cells were co-transfected as indicated with HA-tagged ubiquitin and V5-PDK1. Immunoprecipitation of HA-ubiquitin and input were immunoblotted with a V5 antibody. **D**: HEK293T cells were co-transfected as indicated and whole cell extracts were probed with anti-V5 or anti-HA antibody. **E**: HEK293T cells were transfected with V5-tagged PDK1 or a linear PDK1-ubiquitin C-terminal fusion protein. The whole cell extracts were probed with anti-V5 antibody. **F**: Endogenous PDK1 was immunoprecipitated from HEK293T cells and blotted with anti-PDK1 antibody. The following controls were used: anti-PDK1 antibody only, sepharose beads only, anti-lgG2a control antibody with beads. **G**: Lysates from the indicated cell lines were subjected to PDK1 immunoprecipitation. Immunoprecipitations were immunoblotted with a PDK1 antibody.

To investigate if PDK1 is ubiquitinated, we isolated ubiquitin-modified proteins from HEK293T cells by His-tagged ubiquitin pull-down under denaturing conditions (Figure 1A). Using a PDK1 specific antibody, we observed several distinct bands of which the most abundant migrated approximately 5–10 kDa higher than the major PDK1 isoform. This suggested that PDK1 is modified by a single ubiquitin moiety and the presence of multiple bands is in agreement with previous reports concerning PDK1 splice isoforms [31]. As mono-ubiquitination generally affects protein functionality and therefore may play a role in PDK1 regulation, we wished to explore this observation further. We henceforth refer to the ubiquitin-modified form of PDK1 as Ub-PDK1.

To definitively characterize the observed band as mono-ubiquitinated PDK1, we first expressed V5-tagged PDK1 and performed V5 immunoprecipitation. As expected, immunoblotting with an anti-V5 antibody revealed several PDK1 bands, one of which migrated at the predicted molecular weight of mono-ubiquitinated PDK1 (Figure 1B). Co-expression of HA-tagged ubiquitin and V5-PDK1 further enhanced ubiquitination such that Ub-PDK1 became visible in the whole cell extract (Figure 1C , lower panel) and anti-HA immunoprecipitation confirmed that the slower migrating PDK1 band corresponds to Ub-PDK1 (Figure 1C , upper panel). Furthermore, co-expression of a lysine-less ubiquitin mutant (K0) that cannot mediate chain formation resulted in a similar Ub-PDK1 pattern, further indicating that the observed band is due to a single ubiquitin moiety (Figure 1D). In agreement with this, a linear PDK1-ubiquitin C-terminal fusion protein migrated at the same position in the gel as Ub-PDK1 (Figure 1E).

To determine whether we could detect endogenous Ub-PDK1, we performed PDK1 immunoprecipitation experiments. Immunoblotting with an anti-PDK1 antibody revealed a clear, albeit minor Ub-PDK1 band at the predicted molecular weight (Figure 1F). Importantly, varying levels of endogenous Ub-PDK1 were observed in a variety of cell lines derived from different tumor types (Figure 1G). Together, these experiments indicate that PDK1 mono-ubiquitination is a common and differentially regulated post-translational modification.

### Mono-ubiquitination of PDK1 occurs in the kinase domain

**Figure 2:**
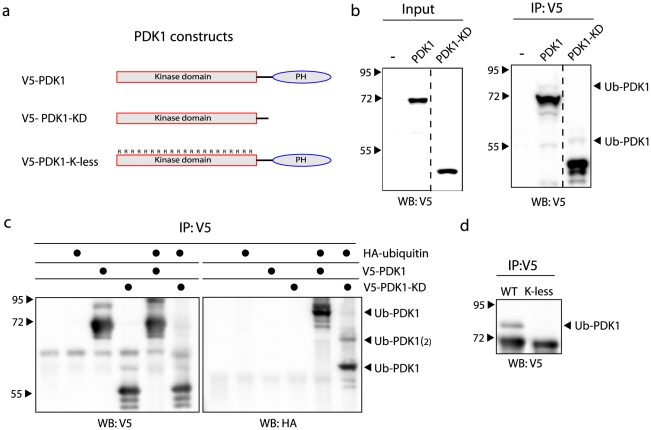
The kinase domain of PDK1 is mono-ubiquitinated. **A**: Overview of the PDK1 constructs used in the figure. PDK1 consists of an amino-terminal kinase domain (KD) and a carboxyl-terminal Pleckstrin Homology (PH) domain. The K-less mutant has all 27 lysine residues (K) mutated to arginines (R). **B**: HEK293T cells were transfected as indicated and PDK1 was immunoprecipitated with anti-V5 beads. Immunoprecipitations (IP) and input were immunoblotted with anti-V5 antibody. **C**: HEK293T cells were transfected as indicated and proteins were immunoprecipitated with anti-V5 beads. Immunoprecipitations (IP) were immunoblotted with V5 and HA antibodies. **D**: HEK293T cells were transfected and the lysine-less mutant of PDK1 was immunoprecipitated with anti-V5 beads and probed with anti-V5 antibody. The wild type construct (WT, V5-PDK1) was used as a control.

To further characterize this novel post-translational modification, we analyzed its site of attachment using a PDK1 deletion mutant (Figure 2A). Cells transfected with full-length PDK1 or just the kinase domain, were analyzed for Ub-PDK1 (a PDK1 mutant lacking the kinase domain was not stably expressed and thus not included in this experiment). A band migrating approximately 5–10 kDa higher than the kinase domain was detectable, suggesting that this domain is ubiquitinated (Figure 2B). Indeed, this was confirmed to be ubiquitinated PDK1 by HA-ubiquitin immunoblot (Figure 2C). In this experiment we detected a second Ub-PDK1 band (labeled Ub-PDK1_(2)_) migrating some 5–10 kDa above the mono-ubiquitinated PDK1, indicating conjugation of two ubiquitin moieties onto a single PDK1 molecule. However, we did not detect conjugation of multiple ubiquitin polypeptides to endogenous PDK1 in the absence of exogenous ubiquitin, suggesting that this only occurs upon overexpression (Figure 1F).

To exclude the possibility that ubiquitin can also conjugate to the PH domain and to further corroborate the observation that ubiquitination occurs in the kinase domain, we mutated all 27 lysine (K) residues of the kinase domain into arginines (R) while keeping the PH domain intact (Figure 2A). As expected, this K-less mutant was not mono-ubiquitinated (Figure 2D). Thus, ubiquitination of PDK1 occurs in the kinase domain.

### Evolutionary conserved lysine residues are not ubiquitinated

**Figure 3:**
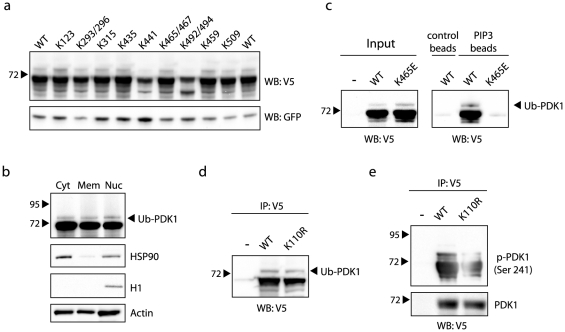
Ub-PDK1 localizes to all cellular compartments, binds phospholipids and mono-ubiquitination is not dependent on kinase activity. **A**: HEK293T cells were co-transfected with wild type V5-PDK1 (WT) or the indicated lysine mutants and GFP. Whole cell extracts were blotted and probed with anti-V5 and GFP that served as a transfection efficiency control. **B**: Cell fractionation analysis of HEK293T cells transfected with V5-tagged PDK1 (Cyt = cytoplasmic, Mem = membrane bound, Nuc = nuclear). Ub-PDK1 was detected with a V5 antibody. The HSP90, Histone H1 (H1) and Actin antibodies were used as controls. **C**: HEK293T cells were transfected as indicated and PDK1 was pulled down using phospholipid-coated (PIP3) or control beads. Pull-downs and input were probed with anti-V5 antibody. **D**: HEK293T cells were transfected and the catalytically inactive mutant K110R of PDK1 was immunoprecipitated with anti-V5 beads and probed with anti-V5 antibody. The wild type construct (WT, V5-PDK1) was used as a control. **E**: HEK293T cells were transfected as indicated and PDK1 was immunoprecipitated with anti-V5 beads. Immunoprecipitations (IP) were analyzed with an antibody recognizing phosphorylated PDK1 at the Ser241 site.

PDK1 comprises 38 lysine residues, each of which could potentially form an isopeptide bond with an ubiquitin molecule. Unfortunately, mutating back each single arginine residue in the K-less mutant to lysine did not restore mono-ubiquitination (not shown). Next, we mutated twelve of the lysine residues conserved between worms, fruit fly, mouse and humans to investigate if any of these would be required for PDK1 mono-ubiquitination. Mutation of K441 and K492/494 resulted in reduced PDK1 protein expression when compared to the GFP transfection control, explaining the reduction in Ub-PDK1 in these samples and indicating that these lysines are not the principal conjugation residues (Figure 3A). Also mutation of the other conserved lysines did not result in a dramatic change of Ub-PDK1. Furthermore, mass spectrometry analysis of purified and trypsin digested Ub-PDK1 was unable to identify the target residue, despite detection of the great majority of predicted tryptic peptides (not shown). A potential explanation for these results may be that PDK1 can be ubiquitinated on various redundant lysine residues, as has been shown for AKT, p53 and Cyclin B, thereby making both the genetic and biochemical approach to identify the site(s) highly challenging [28], [32], [33].

### Ub-PDK1 is nuclear and cytoplasmic, binds phospholipids and mono-ubiquitination is not dependent on kinase activity

PDK1 shuttles between the nucleus and the cytoplasm in a CRM1-dependent and growth factor regulated manner but the functional significance of this remains unclear [13], [14]. As mono-ubiquitination has been reported to regulate subcellular localization, we investigated a potential role for PDK1 mono-ubiquitination in this process. Cells were transfected with PDK1 to enable detection of mono-ubiquitinated PDK1 by immunoblotting, and subsequently fractionated into nuclear, cytoplasmic and membrane fractions. Ub-PDK1 was found to be equally distributed over the nuclear, cytoplasmic and membrane fractions (Figure 3B).

As mentioned, PDK1 is recruited to the plasma membrane via its association with phospholipids such as PIP3, where it is known to function in phosphorylating target proteins. To determine whether PDK1 mono-ubiquitination plays a role in binding to the plasma membrane, we incubated cell lysates from cells transfected with V5-tagged PDK1 with phospholipid-coated beads. The ratio PDK1:Ub-PDK1 in the input and lipid bead bound fraction was very similar (compare lanes 2 and 5), indicating that Ub-PDK1 binds equally well to phospholipid-coated beads as unmodified PDK1. Furthermore, a PDK1 PH domain point mutant that impairs phospholipid binding [34] was still mono-ubiquitinated, indicating that lipid binding is not a pre-requisite for ubiquitination (Figure 3C). Together, these observations indicate that PDK1 mono-ubiquitination is not involved in membrane binding, at least under these conditions.

Next, we determined whether PDK1 mono-ubiquitination is dependent on its kinase activity. A catalytically inactive PDK1 K110R point mutant [35] was found to be comparably mono-ubiquitinated as wild type PDK1 and analysis with a phospho-specific antibody showed that both PDK1 species were phosphorylated, indicating that kinase activity is not required for PDK1 mono-ubiquitination (Figure 3D and 3E).

### USP4 deubiquitinates PDK1 and co-localizes at the plasma membrane

**Figure 4:**
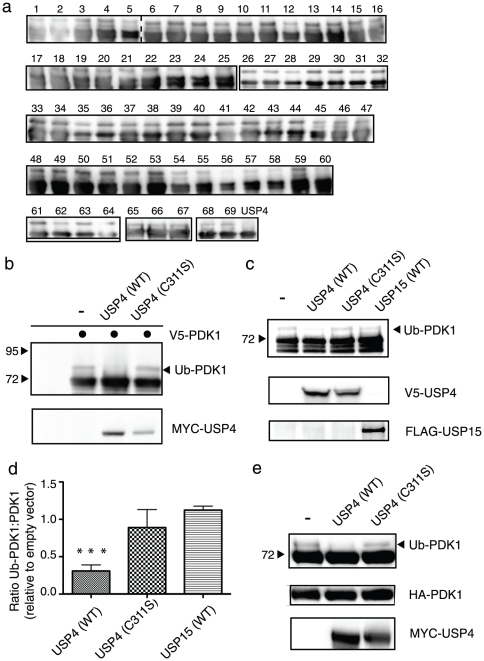
USP4 deubiquitinates PDK1 *in vivo* and *in vitro*. **A**: HEK293T cells were co-transfected with V5-tagged PDK1 and a DUB cDNA library. PDK1 was immunoprecipitated with anti-V5 beads and the Ub-PDK1 was detected using a V5 antibody. **B**: HEK293T cells were co-transfected as indicated. Proteins were immunoprecipitated with anti-V5 beads and immunoblotted with anti-V5 antibody. Whole cell lysates were probed with a MYC antibody to verify the expression of USP4 constructs. **C**: HEK293T cells were transfected with indicated DUBs. Endogenous PDK1 was immunoprecipitated and immunoblotted with a PDK1 antibody. Whole cell lysates were probed with V5 and FLAG antibodies. **D**: Quantification of four independent experiments as in C. Indicated are the mean and standard error of the mean and three asterisks indicate T-test p value<0.001. **E**: HEK293T cells were transfected as indicated and USP4 was immunoprecipitated using a MYC antibody. Immunoprecipitations were incubated *in vitro* with purified HA-tagged PDK1/Ub-PDK1 as a substrate and immunoblotted with anti-HA antibody. The expression of the USP4 constructs was verified with anti-MYC antibody.

As mono-ubiquitination does not lead to protein degradation and can be reverted by deubiquitinating enzymes (DUBs), we screened a total of 70 DUBs for their ability to inhibit Ub-PDK1 upon overexpression (Figure 4A). Tagged PDK1 and each individual DUB were co-expressed in HEK293T cells and analyzed for Ub-PDK1. Although the great majority of DUBs was expressed at high levels (Figure S1), only the Ubiquitin-Specific Protease 4 (USP4) reproducibly inhibited Ub-PDK1 (Figure 4A and 4B). Other candidate hits from the screen, including #24 and #56, failed to show a reproducible reduction in Ub-PDK1 (Figure S2). A catalytically inactive USP4 mutant (C311S), in which the active site cysteine 311 residue is mutated to serine, failed to modulate PDK1 ubiquitination, indicating that the effect of USP4 on PDK1 is dependent on its deubiquitinase activity (Figure 4B). Importantly, the inhibitory effect of USP4 on Ub-PDK1 could also be shown for endogenous PDK1 (Figure 4C and 4D). In addition, the closely related USP15, which shares 61% amino acid sequence identity with USP4 [36], had no effect on Ub-PDK1 levels, further suggesting that the ability of USP4 to deubiquitinate PDK1 is specific.

To investigate if the effect of USP4 on reducing PDK1 ubiquitination could be mediated by direct deubiquitination, we asked if USP4 displayed *in vitro* activity towards Ub-PDK1. Wild type or catalytically inactive USP4 were immunoprecipitated from transfected cells and incubated with purified PDK1/Ub-PDK1. A marked reduction in Ub-PDK1 was evident only in the sample incubated with wild type USP4, indicating that Ub-PDK1 serves as a direct substrate (Figure 4E).

**Figure 5:**
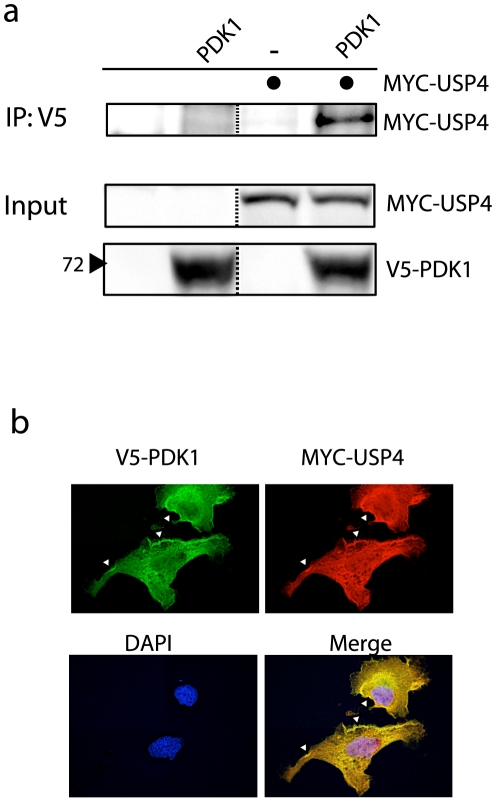
USP4 and PDK1 interact and co-localize at the plasma membrane. **A**: HEK293T cells were transfected as indicated and PDK1 was immunoprecipitated with anti-V5 beads. Immunoprecipitations (IP) and input were probed with anti-V5 and MYC antibodies. **B**: Confocal images from transfected U2OS cells stained with V5-PDK1 (green) and MYC-USP4 (red) antibodies. DNA was visualized by DAPI.

To further investigate a potential role of USP4 in PDK1 deubiquitination, we asked if the two proteins interact upon overexpression. As predicted, MYC-tagged USP4 could be co-immunoprecipitated with V5-PDK1 in HEK293T cells (Figure 5A). A direct effect of USP4 on PDK1 was also suggested by co-localization studies using confocal microscopy. In accordance with previous studies, PDK1 was found to be mainly cytoplasmic with some additional nuclear staining [13], [37]. Interestingly, USP4 and PDK1 co-localized intensely at the plasma membrane when the two proteins were overexpressed (Figure 5B). Taken together, these results identify USP4 as a putative regulator of Ub-PDK1.

## Discussion

We identify a novel post-translational modification, mono-ubiquitination, of PDK1, which plays a central role in signaling via the PI3K pathway to control many cellular processes. We did not obtain evidence that PDK1 ubiquitination is regulated by growth factor signaling. For instance, total Ub-PDK1 did not change upon stimulation with insulin, EGF or glucose under the conditions we tested (not shown). Also cellular fractionation did not reveal a compartment-specific response upon stimulation with insulin (not shown). Nonetheless, it is plausible that PDK1 ubiquitination is modulated under specific conditions or by certain stimuli. Indeed, there is ample evidence that mono-ubiquitination is known to play a crucial role in protein function. For instance, upon DNA damage, the Fanconi Anemia protein FANCD2 is mono-ubiquitinated and re-localizes to the chromatin whereas ubiquitin-modified proliferating cell nuclear antigen (PCNA) recruits a specific DNA polymerase [38], [39].

Furthermore, mono-ubiquitination of tyrosine kinase receptors triggers receptor endocytosis and p53 mono-ubiquitination stimulates its nuclear export [40], [41]. Indeed, future studies will undoubtedly reveal a key regulatory function of PDK1 mono-ubiquitination. This function will be of particular interest as it is not fully understood how PDK1 orchestrates the activation of its many substrates [42]. For instance, PDK1 ubiquitination may modulate binding to adapter molecules and downstream targets or alter subcellular localization. However, the unavailability of a PDK1 point mutant that is not ubiquitinated hampered the investigation of these hypotheses. Indeed, reintroducing single lysine residues in the lysine-less PDK1 mutant did not restore mono-ubiquitination perhaps because mutation of all lysines in the kinase domain precludes interaction with the responsible ubiquitin ligase. The lack of success of the reverse experiment in which conserved lysines were mutated to arginines may be explained by redundancy between target lysines. Perhaps future mass spectrometry experiments with alternative proteases will reveal the ubiquitination site(s) on PDK1 and will enable a systematic functional analysis.

By screening the great majority of DUBs in the human genome, we identified USP4 as the only DUB enzyme that reduced PDK1 ubiquitination. This high degree of specificity makes USP4 a prime candidate as a negative regulator of Ub-PDK1. USP4 has previously been implicated in a number of processes, including protein quality control in the endoplasmatic reticulum and p53 and Wnt signaling [43], [44], [45], [46]. Interestingly, USP4 has been reported to inhibit the kinase TAK1 that is ubiquitinated by the AKT regulator TRAF6. Thus, USP4 may impinge on the PI3K/PDK1/AKT pathway at multiple levels. However, we could not show effects on Ub-PDK1 upon knockdown of USP4, which is possibly explained by functional redundancy with other DUBs or low basal activity of endogenous USP4 (not shown).

In summary, we identified mono-ubiquitination as a novel post-translational modification of PDK1 and propose USP4 as a candidate negative regulator, adding an additional layer of complexity to the PDK1 signaling network.

## Materials and Methods

### Cell lines

The human cell lines were obtained from the American Type Culture Collection. HEK293T (human embryonic kidney cells), HeLa (cervix carcinoma), U2OS (osteosarcoma), HaCat (keratinocytes), A549 (alveolar basal epithelial carcinoma), MCF7 (breast adenocarcinoma), SK-HEP1 and HUH7 (hepatoma) cells were maintained in Dulbecco's modified Eagle's medium. K562 (chronic myelogenous leukemia), A2780 (ovarian carcinoma), THP-1 (acute monocytic leukemia), SNU-387 and HepG2 (hepatoma) were cultured in RPMI-1640 medium. All media were supplemented with 10% fetal calf serum and 1% penicillin/streptomycin at 37°C, 5% CO_2_ and 95% humidity.

### Immunoprecipitation, pull-downs and immunoblotting

For immunoprecipitation and PIP3 (Echelon Biosciences) pull-down, cell extracts were prepared in RIPA buffer (25 mM Tris-HCl (pH 7.6), 150 mM NaCl, 1% Tergitol, 0.5% Triton X-100 and 0.1% SDS) or in ELB buffer (0.1% NP-40, 50 mM Hepes, 250 mM NaCl and 5 mM EDTA) supplemented with protease inhibitors (Complete, Roche) and *N*-Ethylmaleimide (Sigma). The lysate was sonicated for 2×6 seconds on ice, centrifuged at 14.000 rpm for 10 min at 4°C and pre-cleared with 10 µl Protein A/G agarose beads (Pierce #20421). Subsequent immunoprecipitation was performed with 20 µl Protein A/G agarose bead suspension and 1 µg antibody per 1 ml lysate or pre-coupled agarose bead suspension (anti-HA agarose, Sigma A2095; anti-V5, Sigma A7345).

For isolation of His-tagged proteins, cells were lysed under denaturing conditions (10 mM Tris-HCl (pH 8.0), 6 M Guanidine-HCl, 100 mM NaH_2_PO_4_ and 1 mM Beta-Mercaptoethanol) supplemented with *N*-Ethylmaleimide and sonicated. Lysates were incubated for 4 hours with nickel beads (Sigma H0537) and washed with imidazole buffer (10 mM imidazole, 25 mM Tris-HCl (pH 8.0), 150 mM NaCl, 1% NP-40 and 0.1% SDS). Beads were resuspended in sample buffer and boiled for 3 min at 95°C.

For immunoblotting, proteins were resolved by 4–12% Bis-Tris polyacrylamide gels (Invitrogen) and transferred to polyvinylidene difluoride (PVDF) membranes. Membranes were blocked in 2% i-Block (Applied Biosystem) or 4% BSA for 20 min and probed with the appropriate antibody overnight at 4°C. Detection of bound antibodies was performed by incubation with horseradish peroxidase-conjugated anti-rabbit or anti-mouse antibodies at room temperature for 1 h followed by ECL according to the manufacturer's protocol (ECL-PLUS, GE Health Care).

### Antibodies

Anti-PDK1 (sc-17765) and anti-GFP (sc-8334) were purchased from Santa Cruz. Anti-FLAG (F7425), anti-MYC 9E10 (M4439), anti-V5 V5-10 (V8012), anti-HA HA-7 (H9658) and anti-Actin (A2066) were obtained from Sigma. Anti-phospho-specific S241 PDK1 (#3061) and anti-HSP90 (SPS-771) were purchased from Cell Signaling and Stressgen, respectively. Anti-H1.2 (ab17677) was obtained from Abcam.

### Plasmids and cell transfection

The pcDNA6-PDK1-V5-His vector was generated by cloning a PCR fragment with EcoRI and XbaI restriction sites (Fwd-primer 5′GAATTCGCCAGGACCACCAGCCAG3′, Rev-primer 5′TCACTGCACAGCGGCGTCTCTAGA3′) into the pcDNA6 backbone. All other mutants were generated by site-directed mutagenesis using the Stratagene Dpn1 PCR protocol.

The His-tagged ubiquitin expression vector was created by cloning human ubiquitin B into pcDNA3 using the restriction sites BamHI and XbaI. The PDK1-ubiquitin C-terminal fusion protein was created by PCR amplifying human ubiquitin B and cloning it 3′ of PDK1 in pcDNA6-PDK1-V5-His vector using KpnI and EcoRI.

PDK1 kinase domain (KD) expression vector was created by PCR amplification and cloning into pcDNA6-V5-His using EcoRI and XbaI. The following primers were used: Fwd-primer 5′-GATCGAATTCCCACCATGGCCAGGACCACCAGC-3′, Rev-primer 5′-GATCTCTAGACGGGTGAGCTTCGGAGGCGTC-3′.

HA-tagged ubiquitin expression vector (pRK5-HA-ubiquitin wild type) and the 68 DUB expression vectors were obtained from Addgene. cDNAs encoding the full-length USP4-WT and -C311S proteins in the pcDNA4-MYC-His vectors were kindly provided by Dr. Kristina Lindsten and subcloned into pcDNA6-V5-His. The K-less PDK1 mutant was ordered from Mr.Gene (Regensburg, Germany). All generated constructs and mutants were verified by Sanger sequencing.

Cells were transfected using the CaPO_4_ method or with Lipofectamine 2000 (Invitrogen) according to manufacturer's instructions and cells were harvested between 48 and 72 hours post-transfection.

Cell fractionation was performed using the ProteoExtract Subcellular Proteome Extraction Kit (Calbiochem #539791) according to manufacturer's protocol.

### In vitro deubiquitination assay

MYC-tagged USP4 was immunoprecipitated with anti-MYC antibody and co-incubated with purified STREP-HA-PDK1/Ub-PDK1 overnight at 30°C in a final volume for 15 µl of deubiquitination buffer (50 mM Hepes (pH 7.6), 100 mM NaCl, 5 mM MgCl_2_, 5% glycerol, 0.2% Triton X-100, 10 mM DTT and 2 mM ATP). The reaction mixtures were analyzed by immunoblotting with the anti-HA antibody.

### Confocal microscopy

U2OS cells were plated on glass coverslips in 6-well dishes and transfected with 4 µg cDNA using Lipofectamine 2000. After 48 hours, cells were fixed with 4% paraformaldehyde at room temperature for 15 min, permeabilized with 0.1% Triton X-100, blocked with 3% bovine serum albumin-phosphate-buffered saline solution and incubated with anti-V5 (Invitrogen R960-25) or anti-MYC 9E10 (Sigma C3956). After extensive washing, cells were incubated with Alexa-488- (Invitrogen A11001) and Alexa-564-conjugated (Invitrogen A10040) secondary antibodies or DAPI for 1 h. Coverslips were mounted onto slides with ProLong Gold (Molecular Probes). Immunofluorescence-stained cells were visualized with a Leica DMI6000B confocal microscope and images were captured with LAS AF software, version 2.3.0.

## Supporting Information

Figure S1 **The majority of DUB cDNA library is expressed in HEK293T cells.** Cells were co-transfected with V5-tagged PDK1 and a DUB cDNA library. Whole cell lysates were probed with FLAG and MYC antibodies to verify the expression of DUBs.
(PDF)

Figure S2 **Only USP4 shows a reproducible reduction in Ub-PDK1.** HEK293T cells were co-transfected with V5-tagged PDK1 and indicated DUB cDNA clones. PDK1 was immunoprecipitated with anti-V5 beads and the Ub-PDK1 was detected using a V5 antibody. The normalized Ub-PDK1:PDK1 ratio is indicated below each lane. Whole cell lysates were probed with MYC and FLAG antibodies to verify the expression of DUBs. Asterisks indicate unspecific cross-reacting bands.
(PDF)
