# Supplementary figures and images for: Ubiquitin-Specific Protease 4 Inhibits Mono-Ubiquitination of the Master Growth Factor Signaling Kinase PDK1
Source: PLoS One. 2012 Feb 7;7(2):e31003. doi: 10.1371/journal.pone.0031003 (PMC3274522; doi:10.1371/journal.pone.0031003)

Supplementary Figures

S1

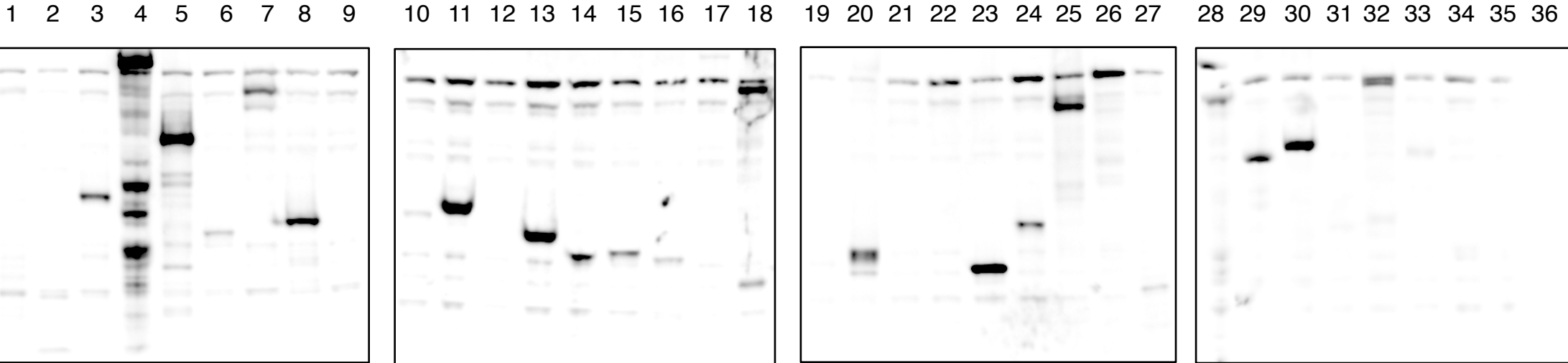

WB: FLAG

WB: FLAG

WB: FLAG

WB: FLAG

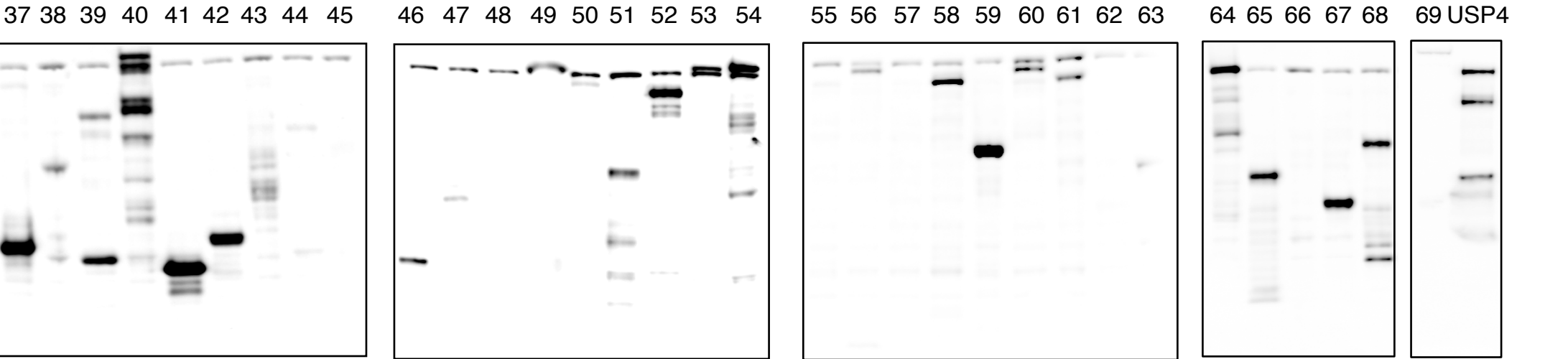

WB: FLAG

WB: FLAG

WB: FLAG

WB: FLAG

WB: MYC

Supplement: Figure S1 — The majority of DUB cDNA library is expressed in HEK293T cells. Cells were co-transfected with V5-tagged PDK1 and a DUB cDNA library. Whole cell lysates were probed with FLAG and MYC antibodies to verify the expression of DUBs. (PDF) [file pone.0031003.s001.pdf]

# Supplementary Figures

S2

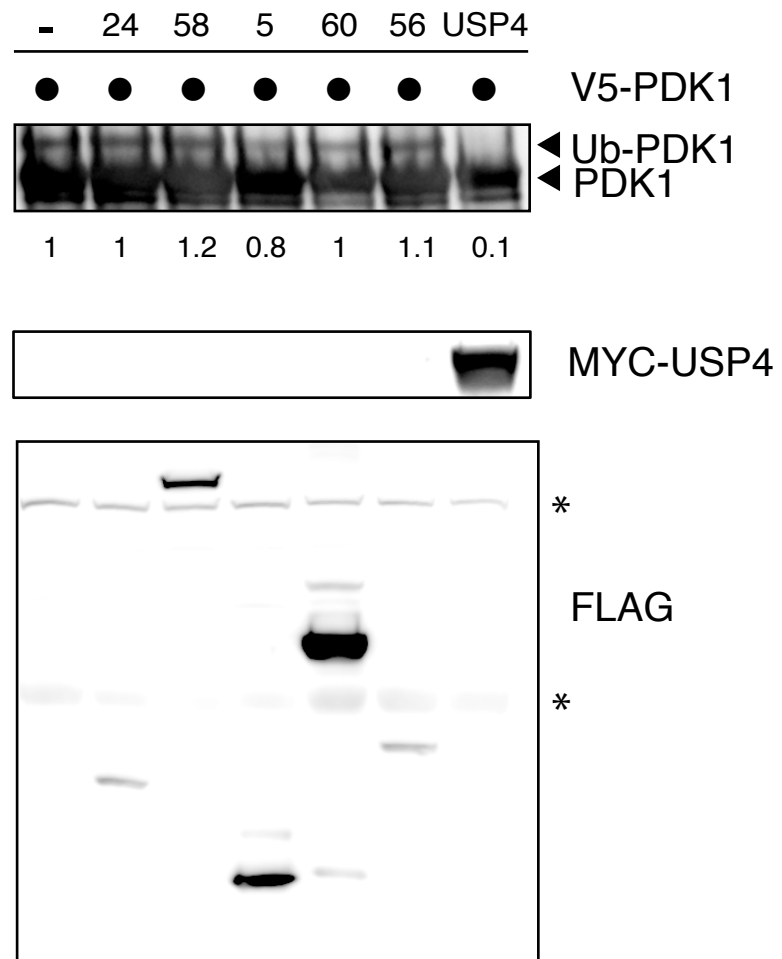

Supplement: Figure S2 — Only USP4 shows a reproducible reduction in Ub-PDK1. HEK293T cells were co-transfected with V5-tagged PDK1 and indicated DUB cDNA clones. PDK1 was immunoprecipitated with anti-V5 beads and the Ub-PDK1 was detected using a V5 antibody. The normalized Ub-PDK1:PDK1 ratio is indicated below each lane. Whole cell lysates were probed with MYC and FLAG antibodies to verify the expression of DUBs. Asterisks indicate unspecific cross-reacting bands. (PDF) [file pone.0031003.s002.pdf]
